# The Influence of *Quercus alba* Geographical Location and Aging Time on the Chemical and Sensory Quality of Tempranillo Wines

**DOI:** 10.3390/molecules29184432

**Published:** 2024-09-18

**Authors:** Zhao Feng, Leticia Martínez-Lapuente, Mikel Landín Ross-Magahy, Manuel Higueras, Belén Ayestarán, Zenaida Guadalupe

**Affiliations:** 1 Instituto de Ciencias de la Vid y del Vino, Universidad de la Rioja, Gobierno de La Rioja y CSIC, Finca La Grajera, Ctra. De Burgos Km 6, 26007 Logroño, Spain; leticia.martinez@unirioja.es (L.M.-L.); milandin@unirioja.es (M.L.R.-M.); belen.ayestaran@unirioja.es (B.A.); zenaida.guadalupe@unirioja.es (Z.G.); 2 Scientific Computation & Technological Innovation Center (SCoTIC), Universidad de La Rioja, 26006 Logroño, Spain; manuel.higueras@unirioja.es

**Keywords:** oak aging, USA forest, phenolic compounds, volatile compounds, ellagitannins

## Abstract

The changes produced during the aging of wines in oak barrels are strongly dependent on the oak’s geographical origin and aging time. This paper analyzes the effect of *Quercus alba* oak from four different geographical locations in four states in the USA, namely Missouri (Mo), Ohio (Oh), Kentucky (Kt), and Pennsylvania (Py), during 24 months of aging. Oak origin had a higher effect on the wine’s aromatic composition than the polyphenolic one. Mo and Oh barrels enhanced coconut, spicy, and sweet notes for 12 months of aging, while Kt barrels achieved higher extraction of wood-related compounds at longer aging (24 months). Py wines showed the lowest contents of most volatile compounds at both aging times, as well as hydroxycinnamic acids, flavanols, anthocyanins, flavonols, stilbenes, and ellagitannins, attributed to their higher porosity. At 12 months of aging, Kt wines showed the highest content of ellagitannins, and Mo wines had the highest content of anthocyanins, but Oh wines had the highest concentrations at 24 months. In the sensory analysis, Kt wines were preferred at both aging times. Kt and Mo wines achieved the highest punctuations for the olfactory phase at 12 months of aging and Kt wines kept it after 24 months. These findings are essential for producers to achieve the sensory characteristics of their wines through strategic barrel aging.

## 1. Introduction

Wine is made up of a complex mixture of chemical compounds, which undergoes various changes during oxidative aging in wooden barrels. Therefore, oak aging considerably affects the volatile composition of the wines, mainly the families including furfural, phenolic aldehydes, volatile phenols, and lactones, which contribute to the wine’s aromatic complexity, imparting notes such as coconut, smoky, toasty, woody, spicy, nutty, and vanilla [1]. In terms of non-volatile substances, oak can provide ellagitannins, hydroxybenzoic acids, and hydroxycinnamic acids, which can affect the astringency, bitterness, and color of the wines due to their chemical reactivity [2,3,4]. Moreover, the porosity of oak allows for moderate diffusion of oxygen inside the barrel, estimated at 10–45 mg/L per year for oak barrels [5]. This oxygenation triggers different reactions of oxidation, polymerization, and condensation involving anthocyanins and tannins, thus stabilizing the color and reducing astringency [6].

In recent years, considerable research has focused on the use of different species of wood (cherry, chestnut, acacia, ash, etc.) [7] or different forms of wood (wood barrel, cask, chips, staves, powders, etc.) [8] for wine aging. Some studies have shown that the high porosity of chestnut and cherry wood provides a more oxidizing environment for wines than oak, making these woods unsuitable for long-term aging [9,10]. In the study by Tavares et al. [11], it was observed that wines aged with French oak chips scored significantly higher on aroma descriptors such as vanilla, boisé, and coconut, while red wines aged with acacia chips and cherry chips showed lower scores for all aroma descriptors. In addition, Rubio-Bretón et al. [12] provided the best organoleptic evaluation of wines macerated with oak staves obtained in a short period of time, revealing that while the organoleptic quality of wines aged in oak barrels improved with time, wines aged in oak chips had the worst sensory ratings at all time.

In this sense, oak (*Quercus* sp.) in the form of barrels still holds a prominent position in the production of high-quality wines. In addition to the traditional species (*Q. petraea* and *Q. alba*), in recent years, *Q. humboldtii* and *Q. pyrenaica* have been found to have an interesting oenological potential as alternatives to traditional oaks [13,14]. It is widely known that the changes produced during the aging of wines in oak barrels depend on the aging conditions such as the size of the barrel, the wood botanical species and its geographical origin, the barrel toasting level, the number of uses of the barrels, and the seasoning [15,16,17,18,19,20], among which the aging time is one of the most important factors [8,21,22].

In Spain, aged red wines are classified according to the storing time in wood barrels and bottles, which provides regulations for the wine industry and helps consumers to better understand the types of wines they are buying and make informed purchasing decisions. The term ‘Crianza’ is used in the Qualified Designation of Origin (D.O.Ca) Rioja to refer to red wines that have been stored in oak barrels and bottles for a minimum of 2 years, with at least 12 months in barrels. The term ‘Reserva’ is applied to red wines that have been stored for at least 36 months, with a minimum of 12 months in oak barrels and 6 months in bottles. Finally, ‘Gran Reserva’ describes red wines that have been stored for 60 months, with a minimum of 24 months in oak barrels and 24 months in bottles. D.O.Ca Rioja has the largest barrel park in the world, reaching 1,372,280 barrels in 2021 (Regulatory Council of the Rioja Qualified Denomination of Origin, 2021). Furthermore, according to available data from 2018 to 2021, over 65% of D.O.Ca Rioja red wine sold in the market was aged in oak barrels. During this period, the production of ‘Gran Reserva’ red wines continued to grow, reaching 7,943,985 L in 2021, which accounted for 5.5% of the total production of aged red wines in the same year (Regulatory Council of the Rioja Qualified Denomination of Origin, 2018–2021).

The effects of aging in oak barrels for long periods (12–21 months) on the aromatic, phenolic, and organoleptic characteristics of red wines have been described by Fernández de Simón et al. [23,24], and Garde-Cerdan et al. [25]. However, the number of studies that analyze the aging time in barrels for periods of two years is very limited. Guld et al. [26] studied the effect of time during the aging of red Hungarian wines in Hungarian oak barrels (*Quercus petraea*) and observed an increasing trend in *trans*-piceid and *trans*-resveratrol concentrations and a decrease in anthocyanin concentration over time. Castro-Vázquez et al. [27] studied the evolution of volatile compounds in Tempranillo wines during 36 months of barrel aging and observed that the concentration of C6 alcohols, volatile acids, and terpenes decreased to different extents during aging, while the concentration of β-methyl-γ-octalactone isomers, guaiacol, eugenol, syringol, vanillin, and ethyl vanillate increased, as aging progressed. Michel et al. [21] concluded that the polyphenol index of French oak wood affected the concentration and evolution of ellagitannins in the wine, with very different concentrations ranging from 2.30 to 32.56 mg/L at the end of 24 months of aging, in agreement with the classification of *Q. petraea* wood from the lowest to the highest polyphenol index. From a sensorial aspect, French oak barrels (*Quercus petraea*) were described to be more suitable for the short aging (6–12 months) of Tempranillo wines, while barrels made of American oak (*Quercus alba*) were found to be more suitable for longer aging (18–24 months) because the sensory quality of the wines aged in *Q. alba* improved as the aging time increased [28]. Recent studies of our group [29,30] revealed that the forest origin of *Q. alba* barrels has a clear influence on the chemical and sensory quality of aged Tempranillo wines. The oak origin affected the color parameters of aged wines, the total polyphenol index, and the phenolic composition. Wines aged in Pennsylvania barrels exhibited the lowest content of phenolic compounds and color intensity after 12 months of aging, while wines aged in Kentucky and Ohio barrels showed the highest content of ellagitannins [30]. Oak barrels from the forests of Missouri and Pennsylvania produced wines with the highest aromatic compounds related to floral, fruity, and wood-related aromas, and the greatest aroma complexity after 6 months of aging, but these differences decreased after 12 months of aging, and the wines aged in Missouri and Kentucky barrels were the ones with the highest OAV and best valued in the sensory analysis. The results obtained in these studies aim to help producers choose the type of barrel to age their wines depending on the characteristics they want to achieve and the aging time of their wines. Considering the long aging times of quality wines in Spain, it is essential to know the influence of these origins in longer periods of time, at least 24 months of aging, corresponding to the ‘Gran Reserva’ wines, to determine if the differences among origins are maintained or change over time. Therefore, the aim of this study was to investigate the effect of *Q. alba* oak from four different geographical locations in the United States (Missouri, Ohio, Kentucky, and Pennsylvania) on the volatile, phenolic composition, and sensory perception of Tempranillo wines aged for 12 and 24 months.

## 2. Results and Discussion

### 2.1. Common Oenological Parameters

Table 1 shows the general composition (pH, total and volatile acidity, alcoholic strength, color intensity, total phenolic index, and tonality) of the Tempranillo wines after 12 and 24 months of aging in oak barrels. The results of common oenological parameters revealed the correct development of aging treatment. All values agreed with the normal content found in red wines after barrel aging [31,32,33]. Wines showed significant differences in TPI values at both aging times, and significant differences in CI were observed after 24 months of aging, mainly due to a decrease in the red color component at 520 nm but also at 420 nm. The tonality of the wine (as absorbance at 420 nm/absorbance at 520 nm) increased during the aging process mainly due to a decrease in absorbance at 520 nm. Wines aged in Mo barrels showed the highest TPI values in both aging times, while wines aged in Oh barrels exhibited the highest CI values at 24 months of aging. A decrease in the color intensity was observed in all the wines during the aging period in oak barrels. These results could be related to the loss of total anthocyanin content during the aging period (Table 2 and Table 3). Regarding the TPI, there was also a decrease throughout the aging for all wines due to the precipitation of a proportion of polyphenols. In fact, from 12 to 24 months of aging, TPI levels decreased by 14%, 19%, 17%, and 15% in wines aged in barrels Kt, Mo, Oh, and Py, respectively (Table 1). In agreement with Kyraleou et al. [34], TPI progressively decreased during aging, possibly due to the transformation of phenolic compounds into more condensed chemical constituents with different properties and reactivity. Flavanols can polymerize with each other and with anthocyanins to form a flavanol-ethyl–flavanol adduct or a flavanol–ethylanthocyanin adduct. These complexes can participate in additional acetaldehyde-mediated polymerizations with other flavanols or anthocyanins in wines, and when the complexes are too large, they may precipitate [35].

### 2.2. Volatile Composition and Odor Activity Values of Wines after 12 and 24 Months of Aging

The concentration of wood-related volatile compounds and the odor activity values (OAV) of the wines after 12 months of aging are shown in Table 4 and Table 5, respectively. Similarly, Table 6 and Table 7 show the concentrations of wood-related volatile compounds and the odor activity values in wines after 24 months of aging, respectively.

After 12 months of aging (Table 4), wines aged in *Quercus alba* barrels of different geographical origins exhibited significant differences in the concentrations of wood-related volatile compounds. Wines aged in Kt barrels showed the lowest concentration of 4-ethylphenol and the highest of furfural, 5-methylfurfural, and furfuryl alcohol. Kt and Mo wines showed the highest content of eugenol. Wines aged in Mo and Oh barrels presented the highest concentration of *cis*-β-methyl-γ-octalactone. The wines from Oh barrels showed the highest content of guaiacol. Wines from Py barrels showed the highest content of 4-ethylguaiacol and *trans*-β-methyl-γ-octalactone. Aged wines did not show significant differences in the content of vanillin. In general, these results agreed with those in our previous published work [29], which analyzed the volatile composition of Tempranillo wines from 12 different wineries after 12 months of aging in barrels of different origins.

Among wood-related aromas, for four compounds, we found OAV > 1 in all wines after 12 months of aging, namely *cis*-β-methyl-γ-octalactone, providing notes of woody, coconut, and vanilla; eugenol, providing notes of clove, honey, and spice; guaiacol with smoke, toasted, and spicy notes; and vanillin, with vanilla notes (Table 5). As observed by Feng et al. [29], Kt and Mo wines showed the highest OAV of eugenol after 12 months of aging. Mo and Oh wines showed the highest OAV of *cis*-β-methyl-γ-octalactone, and Oh wines also showed the highest OAV of guaiacol. According to the obtained results, to age wines in barrels for 12 months, it would be advisable to use Mo and Oh barrels to enhance the aromatic complexity of the wines with coconut and spicy notes.

Volatile compound concentrations underwent considerable changes from 12 to 24 months of aging (Table 4 and Table 6). Furfural, 5-methylfurfural, and guaiacol concentrations decreased in all wines from 12 to 24 months of aging. Furfural levels decreased by 85%, 82%, 86%, and 80%, in wines aged in barrels Kt, Mo, Oh, and Py, respectively. Similarly, 5-methylfurfural levels decreased by 86%, 73%, 88%, and 85%, in wines aged in barrels Kt, Mo, Oh, and Py, respectively. Guaiacol concentrations decreased by 26%, 11%, 46%, and 24%, in wines aged in barrels Kt, Mo, Oh, and Py, respectively. Castro-Vázquez et al. [27] also observed a decrease in furfural concentrations after aging periods of 24 months or higher, due to its transformation in furfuryl alcohol, which is an aging time marker compound for ‘Gran Reserva’ wines. As observed in previous studies [43,44], the content of guaiacol decreased during aging, which on the one hand could be due to the fact that guaiacol in the wine and the oak barrel existed in a dynamic equilibrium, allowing it to be adsorbed onto the surface of the oak barrel, while on the other hand, during the aging process, guaiacol could interact with anthocyanins to form polymeric pigments, resulting in its reduction [45]. Furfuryl alcohol, eugenol, 4-ethylguaiacol, 4-ethylphenol, and vanillin were extracted constantly in wines during oak aging, indicating the importance of wood contact time. The greatest increase was observed in 4-ethylguaiacol, which increased by 262%, 97%, 233%, and 130%, in wines aged in barrels Kt, Mo, Oh, and Py, respectively. The concentrations of ethylphenols found in wines aged in barrels at the end of the process did not exceed those considered harmful to the wine aroma, 140 and 620 µg/L for 4-ethylguaiacol and 4-ethylphenol, respectively [41]. In the case of β-methyl-γ-octalactones, differences were observed among the wines according to the origin of the wood. An increase of 34% and 17% was observed in wines aged in barrels Kt and Py, respectively, while a decrease of 14% and 6% was observed in barrels Mo and Oh, respectively. These differences can be attributed to the dynamic chemical behavior of β-methyl-γ-octalactones and interactions within the wine matrix. When these lactones are extracted from the oak wood into the wine, they are subject to various chemical transformations to produce the corresponding acids and ethyl esters [46]. The concentration of β-methyl-γ-octalactones in the wine is governed by the balance between their extraction rate from the oak and their degradation rate within the wine.

After 24 months of aging (Table 6), wines aged in Oh and Py barrels showed the lowest concentrations of furfural, 5-methylfurfural, and eugenol. Furfural and 5-methylfurfural are involved in the condensation of phenolic compounds [47,48,49]. A higher oxygen permeability of Oh y Py barrels would explain a higher condensation and polymerization in the wines aged in these barrels and thus a lower content of furfural and 5-methylfurfural in wines aged in these barrels. This higher permeability would lead to more oxidation reactions of eugenol, explaining its lower content [50]. Kt and Oh wines had the largest number of volatile wood compounds with the highest concentration (furfuryl alcohol, *trans*-β-methyl-γ-octalactone, and *cis*-β-methyl-γ-octalactone). The Kt wines also showed the highest values of eugenol. Mo wines showed the highest contents in 5-methylfurfural and Py wines, in 4-ethylphenol. No significant differences were observed in the content of guaiacol after 24 months of aging (Table 6). In both aging times (Table 4 and Table 6), Py wines showed the lowest concentrations of furfural, 5-methylfurfural, *cis*-β-methyl-γ-octalactone and eugenol, while the content of furfural, furfuryl alcohol, and eugenol was significantly higher in Kt wines. Likewise, wines aged in Oh barrels had higher values of *cis*-β-methyl-γ-octalactone and 4-ethylphenol.

Similar to the results obtained at 12 months of aging (Table 5), *cis*-β-methyl-γ-octalactone, eugenol, guaiacol, and vanillin showed values above their perception threshold in all the wines with 24 months of aging (Table 7). Kt and Mo barrels showed the highest OAV of eugenol at both 12 and 24 months of aging, indicating that these barrels provide a clove-like aroma for short and long periods of aging. Wines aged in Py barrels showed the lowest total OAV values for the wood-related aromas in both aging times. However, the OAV of Tempranillo wines aged in different oak barrels exhibited distinct patterns over time for other volatile compounds. In fact, in contrast to what was observed at 12 months of aging, wines from Kt and Oh at 24 months of aging showed the highest OAV of *cis*-β-methyl-γ-octalactone, while wines aged in Kt barrels showed the highest total OAV values for the wood-related aromas, followed by Mo and Oh wines.

Therefore, these results suggest that the specific origin of the oak provided different aromatic profiles depending on the time of aging. For 12 months of aging, Mo and Oh barrels could be recommended to enhance spicy and sweet notes, while for 24 months of aging, Kt barrels could be the preferred choice to achieve a rich and complex aromatic profile. Py barrels provided the lowest total OAV values in both aging times. These findings are essential for winemakers to achieve sensory characteristics in their wines through strategic barrel aging.

### 2.3. Phenolic Composition of Wines after 12 and 24 Months of Aging

Table 2 and Table 3 show the phenolic content of wines aged in Kt, Mo, Oh, and Py oak barrels for 12 and 24 months, respectively. The wines aged in the different origins showed fewer differences in their phenolic composition than in their volatile composition, indicating that the origin of the oak had a more marked influence on the aromatic composition of the wines than the polyphenolic one.

After 12 months of aging (Table 2), wines aged in Kt and Mo oak showed higher concentrations of ellagitannins and total anthocyanins, respectively. By contrast, wines aged in Py barrels showed the lowest concentration of total hydroxybenzoic acids and ellagitannins, hydroxycinnamic acids, flavonols, stilbenes, and anthocyanins, probably because oak staves from Py origin have a finer grain and thus are more permeable to oxygen, which accelerates the oxidation of red wine phenolics. These results agreed with those obtained in previous studies [30], which analyzed 12 different Tempranillo wines aged for 12 months.

After 24 months of aging (Table 3), Py wines maintained the lowest concentration of hydroxycinnamic acids, flavanols, anthocyanins, flavonols, stilbenes, and ellagitannins. Kt wines showed the highest concentration of stilbenes, and Oh wines showed the highest concentration of flavonols, ellagitannins, and anthocyanins, in agreement with its highest values in CI after 24 months of aging (Table 1). No significant differences were observed in the rest of the phenolic families.

Regarding individual phenolic compounds, Py wines showed the lowest contents of gallic and ellagic acids at 12 months of aging (Table 2). Wines aged in oak barrels for 24 months (Table 3) did not show significant differences in the concentrations of gallic acid and syringic acid, and Oh wines showed the lowest concentration of ellagic acid. Oak barrels can provide hydroxybenzoic acids to wines because they are components of the wood [51,52]. Moreover, gallic acid and ellagic acid can also be produced by the hydrolysis of hydrolyzable tannins in oak [53], and syringaldehyde in oak can be oxidized to syringic acid [51]. Finally, these compounds may undergo adsorption onto the surface of the wood, enzymatic or chemical degradation, or sorption onto new products resulting from the polymerization of wine, making it difficult to detect clear trends [54].

Except for hydroxybenzoic acids, wines aged in Py barrels showed the lowest content of most phenolic compounds at both aging times (Table 2 and Table 3). Hydroxycinnamic acids were present in the wine mainly in the esterified form with tartaric acid (caftaric, coutaric, and fertaric acids), and their corresponding free form (caffeic, coumaric, and ferulic acids) were present in comparatively lower concentrations. Py wines showed lower concentrations of most hydroxycinnamic acids at both aging times (Table 2 and Table 3), attributed to a higher oxygen permeability of Py oak barrels, which led to the oxidation of these compounds and thus to a decrease in their concentration [12,33].

Py wines also showed a lower concentration of most flavonols, again attributed to greater oxidation and condensation reactions in wines aged in Py barrels [55]. Chinnici et al. [56] observed that the stronger oxidative environment of cherry wood resulted in a greater decrease in flavonols in wine aged in cherry barrels compared to wine aged in oak barrels. 

Regarding flavan-3-ols, Py wines showed again the lowest content of catechin at both aging times, which was attributed to the participation of catechin in oxidation reactions and its polymerization and condensation with anthocyanins [12,57], again related to the continuous diffusion of oxygen in the barrels [12,57]. Martínez-Gil et al. [57] also found that wines aged in LOTR (low oxygen transmission rate) oak barrels exhibited higher concentrations of catechin compared to those aged in HOTR (high oxygen transmission rate) oak barrels, possibly due to increased oxygen supply in the latter, leading to more reactions involving catechin. Epicatechin was not detected in any wine at 12 months of aging, but it was detected after 24 months of aging, which was attributed to the hydrolysis of higher oligomers [57]. Zhang and Wang [58] and Gutiérrez et al. [59] also observed an increase in the concentration of this compound during the aging process. Py wines again showed the lowest concentration of epicatechin after 24 months of aging. During the aging process, trans-piceid can undergo hydrolysis, releasing free resveratrol [33]. Additionally, resveratrol can be adsorbed onto the surface of the oak [60] and can also participate in oxidation reactions [61]. Py wines showed the lowest content of *trans*-resveratrol compounds, possibly due to their higher porosity leading to the oxidation of resveratrol. 

Finally, the wines aged in Py barrels showed the lowest contents of ellagitannins and all anthocyanin compounds. The lower content of ellagitannins was attributed to both a lower release of these compounds in Py barrels and a higher oxygen permeability of these barrels, which may lead to a more oxidative reaction of ellagitannins. Ellagitannins in wine are exclusively sourced from oak wood. Their concentration in wine affects the taste and color of the wine and is therefore used as one of the most important parameters in the selection of oak barrels for aging [16,62]. Previous studies have also revealed ellagitannin content variations according to oak species and their geographical origin [62,63]. The lowest content of anthocyanins in Py barrels was again attributed to its higher oxygen permeability, leading to oxidation, condensation, and polymerization reactions [57,64,65].

In conclusion, aging in Py barrels produced wines with the lowest contents of most phenolic compounds at both aging times. However, in relation to the highest contents, the same was not observed at 12 and 24 months of aging. At 12 months of aging, Kt wines showed the highest content of ellagitannins, and Mo wines had the highest concentration of anthocyanins. After 24 months of aging, wines aged in Oh barrels showed the highest concentrations of flavonols, ellagitannins, and anthocyanins, indicating that this barrel is more appropriate for achieving greater extractions in long aging times.

### 2.4. Sensory Evaluation of the Wines after 12 and 24 Months of Aging

**Figure 1:**
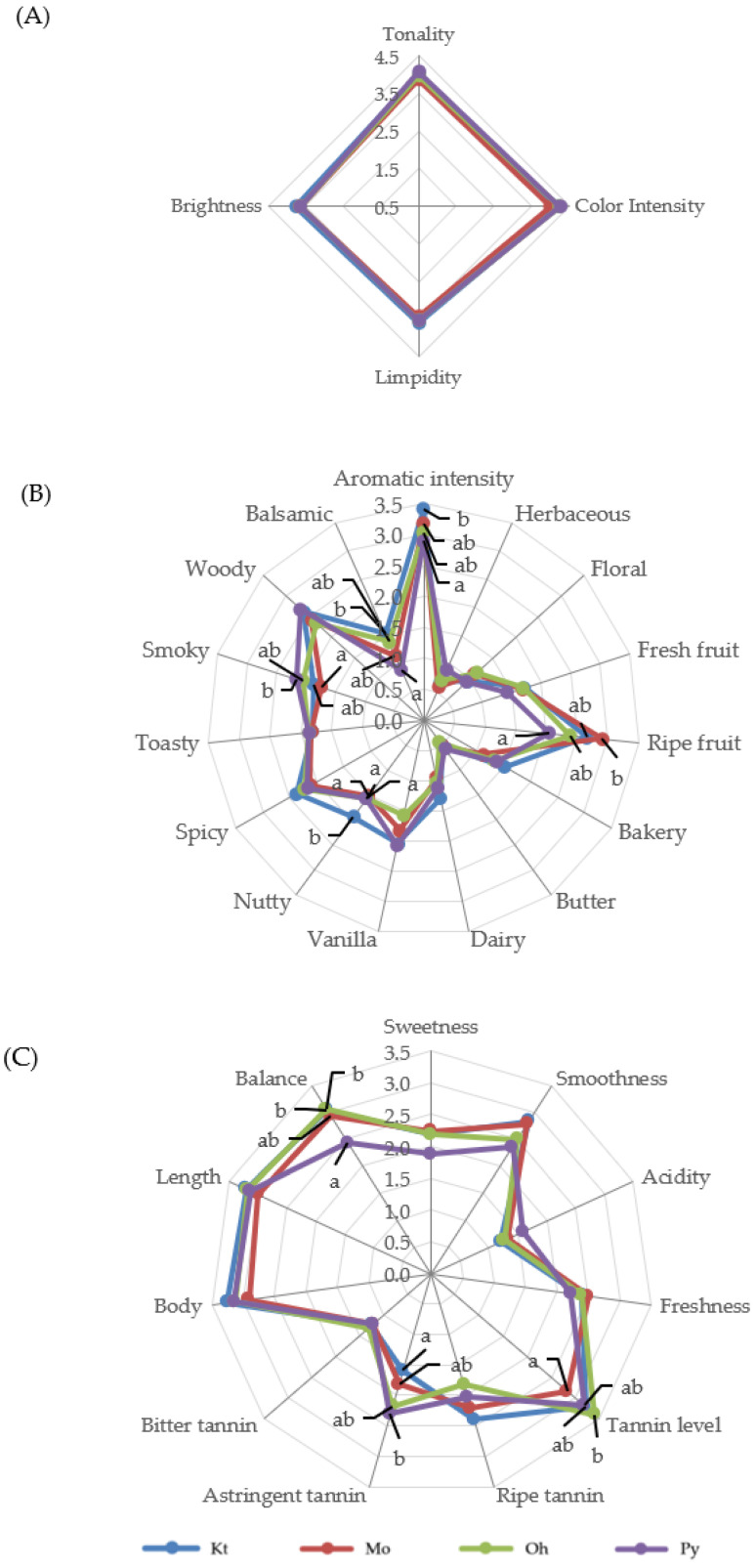
Visual (**A**), olfactory (**B**), and gustatory (**C**) phases of sensory analysis of Tempranillo wines aged for 12 months in oak barrels from Kentucky (Kt), Missouri (Mo), Ohio (Oh), and Pennsylvania (Py). Different letters on the same line indicate statistically significant differences (*p* < 0.05).

**Figure 2:**
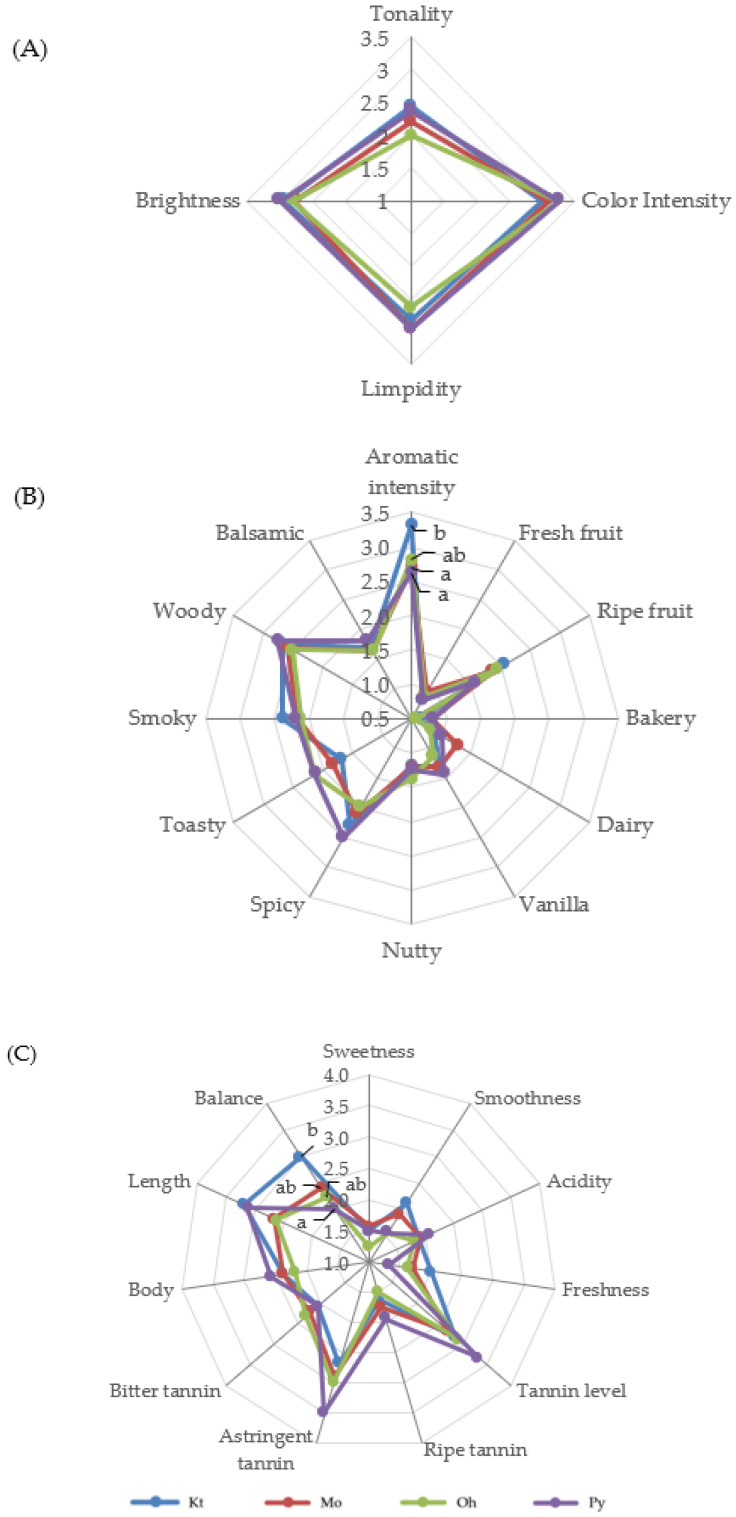
Visual (**A**), olfactory (**B**), and gustatory (**C**) phases of sensory analysis of Tempranillo wines aged for 24 months in oak barrels from Kentucky (Kt), Missouri (Mo), Ohio (Oh), and Pennsylvania (Py). Different letters on the same line indicate statistically significant differences (*p* < 0.05).

Figure 1 and Figure 2 show the average scores of the visual, olfactory, and gustatory descriptors of the Tempranillo wines aged in barrels after 12 and 24 months, respectively. The geometric mean (GM%) was calculated for each attribute according to ISO 11035 [66] to make possible to eliminate the descriptors with low GM. The olfactory attributes of phenolic, reduction, chemical, animal, mineral, and oxidation in wines aged for 12 and 24 months, as well as herbaceous, floral, and butter in wines aged for 24 months, showed GM values lower than <15% and were thus eliminated. The rest of the sensory attributes were used to define the sensory properties of wines.

Wines aged in barrels of different origins did not show significant differences in the visual phase in any aging time (Figure 1A and Figure 2B). Wines at 12 months of aging were characterized by high color intensity, limpidity, and brightness (Figure 1A). Both the visual color intensity and tonality decreased from 12 to 24 months of barrel aging, in good agreement with the data obtained for the enological parameters (Table 1) and the phenolic composition of the wines (Table 2 and Table 3). The tasting panel also evaluated the global perception of the visual phase. Wines aged for 12 months in Kt, Mo, Oh, and Py oak barrels obtained scores of 4.13, 3.76, 4.04, and 3.94, respectively, with no significant differences among them. After 24 months of aging, Kt, Mo, Oh, and Py wines scored 3.44, 3.15, 3.06, and 3.38, respectively, again showing no significant differences.

In the olfactory phase, wines aged for 12 months showed a high aromatic intensity, with high scores for ripe fruit and woody descriptors and relatively low scores (<1) for herbaceous, floral, and butter aroma descriptors (Figure 1B). Concerning non-wood-related aromas, the wines exhibited significant differences only in the descriptors of ripe fruit and balsamic. Wines aged in Kt and Mo barrels exhibited higher punctuations for balsamic and ripe fruit aromas, respectively. Regarding wood-related aromas, a nutty (toasted almonds) aroma, attributed to furanic compounds, was perceived with greater intensity in the wines from Kt barrels, in agreement with the analytical data, as wines aged in Kt barrels for 12 months showed the highest concentration of furanic compounds (Table 4). Py wines achieved a higher score in smoky aromas, which are related to guaiacol and 4-ethylguaiacol, also in good agreement with the analytical data and OAV values (Table 4 and Table 5). Kt wines showed the highest aromatic intensity. Conversely, Py wines showed the lowest punctuations for aromatic intensity and also showed the lowest OAV values for wood-related aromas (Table 5). No significant differences were observed among the wines in the descriptors of vanilla, spicy, toasty, and woody aromas. In the overall perception of the olfactory phase, Kt and Mo wines received the highest scores (3.12 and 3.13, respectively), followed by the wines aged in Oh barrels (3.05) and those aged in Py barrels (2.77). Wines aged for 24 months (Figure 2B) showed an appropriate aromatic intensity, with relatively high scores for woody attributes and relatively low scores (<1) for fresh fruit and bakery aroma descriptors. Wines aged in barrels of different origins only showed significant differences in the descriptor aromatic intensity, with the Kt wines considered the ones with the highest aromatic intensities and the Py ones with the lowest. These results are consistent with those obtained for aromatic intensity at 12 months of aging (Figure 1B), and it is also important to point out that Kt wines showed the highest OAV values for wood-related aromas, whereas Py exhibited the lowest (Table 7). In the global perception of the olfactory phase, Kt wines again achieved the highest scores (3.00), whereas Py wines had the lowest (2.56).

In the gustatory phase, wines aged for 12 months (Figure 1C) were characterized by high values of smoothness, tannin level, body, length, and balance. Significant differences were found in the descriptors of tannin level, astringent tannins, and balance. The panelists perceived a higher balance in Kt, Mo, and Oh wines, and the lowest balance in Py wines. It is possible that the lower levels of flavan-3-ols and ellagitannins in Py wines resulted in their lower scores on the balance descriptor. With respect to the global perception of the gustatory phase, Oh, Mo, and Kt wines did not show significant differences (3.31, 3.29, and 3.19, respectively), while Py wines showed the lowest values (2.86). Regarding the gustatory phase at 24 months of aging (Figure 2C), wines showed high values of tannin level, astringent tannin, length, and balance. These wines only showed significant differences in the balance descriptor. Kt wines were perceived as the most balanced, while Py wines again showed lower values on this descriptor. Concerning the overall perception of the gustatory phase, the scores for wines Kt, Mo, Oh, and Py were 3.06, 2.81, 2.85, and 2.38, respectively, with only Kt and Py wines showing a significant difference between them. 

**Figure 3:**
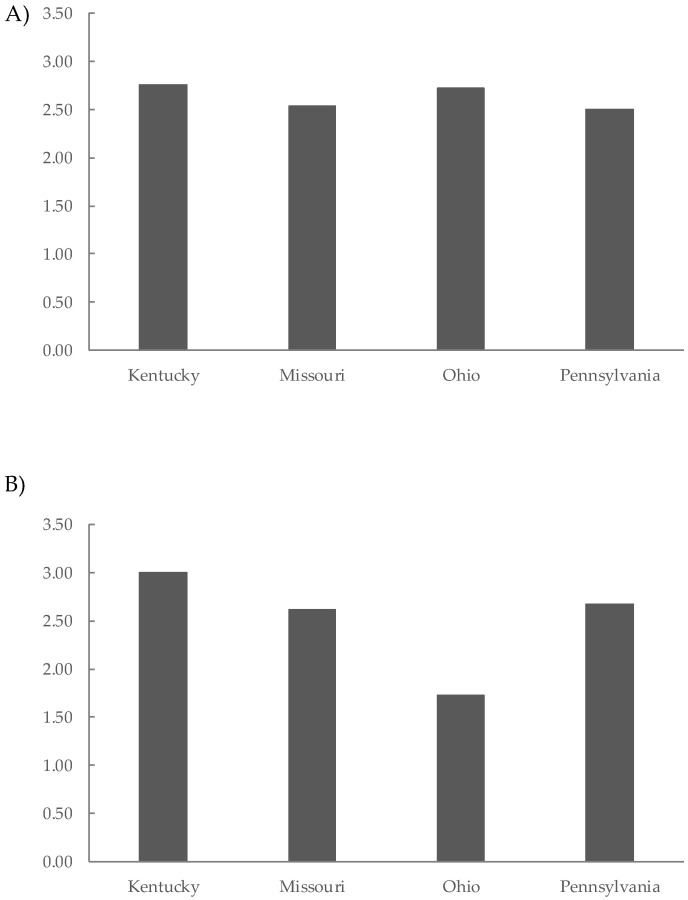
Preference sensory test of Tempranillo wines aged for 12 months (**A**) and 24 months (**B**) in oak barrels from Kentucky, Missouri, Ohio, and Pennsylvania.

Finally, the results of the preference sensory test at 12 and 24 months of aging are shown in Figure 3. The highest scores correspond to the most preferred wines. At 12 months of aging (Figure 3A), the Kt wines were the most preferred (2.75), followed closely by the Oh wines (2.71), Mo wines (2.54), and Py wines (2.50). At 24 months of aging, the preference rankings still indicated that the Kt wines had the highest score at 3.00, followed by Py, Mo, and Oh wines (Figure 3B). These results highlight the importance of selecting an appropriate barrel forest origin for wine aging to optimize wine quality and consumer satisfaction.

## 3. Materials and Methods

### 3.1. Barrels

All oak barrels were constructed by Murua Cooperage (Logroño, La Rioja, Spain) in 2018 using staves of *Q. alba* oak from four different forests in the United States: Missouri, Kentucky, Ohio, and Pennsylvania. These barrels were new with a capacity of 225 L and medium-intensity-toasted, as previously described in Feng et al. [29,30].

### 3.2. Barrel Aging and Sample Collection

Vinifications were made in 3 wine cellars from D.O.Ca Rioja using *Vitis vinifera* cv Tempranillo by traditional red winemaking as described in Feng et al. [29,30]. After malolactic fermentation, wines were aged in triplicate in four different geographical origins of *Q. alba* oak barrels for a period of 24 months. During the two years of aging, the cellar’s humidity ranged from 70 to 75%, and the temperature ranged between 14 and 16 °C [29,30]. Considering that 3 wineries participated in this study, a total of 36 barrels were used. Wine samples were collected at the end of 12 and 24 months of aging and were named Kt, Mo, Oh, and Py based on the barrel origin (Missouri, Mo; Kentucky, Kt; Ohio, Oh; and Pennsylvania, Py).

### 3.3. Analysis of General Oenological Parameters

The general oenological parameters were analyzed in wines after 12 and 24 months of aging according to the OIV methods [67]. Color intensity (CI) and tonality were analyzed using the OIV methods [67]. The total polyphenol index (TPI) was determined by measuring the absorbance at 280 nm [68].

### 3.4. Analysis of Monomeric Phenolic Compounds

Flavonols, flavan-3-ol, hydroxybenzoic acids, hydroxycinnamic acids, stilbenes, and anthocyanins were analyzed by high-performance liquid chromatography (HPLC) (Agilent Technologies, Palo Alto, CA, USA) with photodiode array detection (DAD). Prior to the analysis, the wines were filtered through 0.45 µm membranes, and 25 µL samples were directly subjected to HPLC-DAD. A LiChrospher 100 RP-18 (Agilent) column with a 5 μm particle size (250 mm × 4 mm) was used to separate the analytes [69]. Phenolic compounds were eluted under the following conditions: 1 mL/min flow rate; solvent A: 50 mmol/L NH_4_H_2_PO_4_, pH = 2.6; solvent B: 80% acetonitrile + 20% solvent A; and solvent C: 200 mmol/L o-phosphoric acid, pH = 1.5. The following gradient was used: isocratic 0% B and 0% C during 5 min, from 0% to 8% B in 12 min, from 8% to 14% B and 0% to 86% C in 5 min, from 14% to 18% B and 86% to 82% C in 7 min, from 18% to 21% B and 82% to 79% C in 26 min, from 21% to 33% B and 79% to 67% C in 15 min, from 33% to 50% B and 67% to 50% C in 8 min, from 50% to 80% B and 50% to 0% C in 8 min. The quantification of phenolic compounds was performed as described by Feng et al. [30]. All analyses were performed in triplicate.

### 3.5. Analysis of Ellagitannins

The method described by Peng et al. [70], with some modifications, was used to determine the total ellagitannin content (as mg/L of castalagin) using HPLC-DAD after acid hydrolysis. Modifications to the method of Peng et al. [70] have been described in the study by Feng et al. [30]. Separation was performed using an Agilent XDB-C18 chromatographic column (150 × 4.6 cm × 5 µm), with detection at 370 nm.

### 3.6. Determination of Volatile Compounds and Odor Activity Values

The volatile compounds in wines were analyzed according to Oliveira et al. [71], with modifications based on Coelho et al. [72].

A volume of 3 µg of internal standard (4-nonanol) was added to 8 mL of wine with a magnetic stirring bar. The sample was agitated with 400 µL of CH_2_Cl_2_ to extract the volatiles. After cooling at 0 °C for 10 min, the organic phase was separated by centrifugation (5118 g, 5 min, 4 °C) and was transferred into a vial. The aromatic extract (200 μg/L) was dried using anhydrous sodium sulfate and transferred to a new vial [72]. The extraction of volatile compounds was realized in triplicate for each wine. Analysis was performed by gas chromatography–mass spectrometry (GC-MS) using an Agilent GC 7890 N chromatograph (Agilent Technologies, Palo Alto, CA, USA) coupled with a mass spectrometer Agilent 7000 C. A 1 μL injection was carried out using a capillary column coated with CP-Wax 52 CB (50 m × 0.25 mm i.d., 0.2 μm film thickness, Chrompack, São Paulo, Brazil). The injector temperature was set between 20 °C and 250 °C at a rate of 180 °C/min. The oven temperature was initially held at 40 °C for 5 min and then raised from 40 °C to 250 °C at a rate of 3 °C/min, held at 250 °C for 20 min, and finally programmed to rise from 250 °C to 255 °C at 1 °C/min. The carrier gas was helium N60 (Air Liquide) at a pressure of 103 kPa, corresponding to a linear speed of 180 cm/s at 150 °C. The detector operated in electronic impact mode (70 eV) with an acquisition range of 29 to 360 m/z and an acquisition rate of 610 ms [71]. The identification of the volatile compounds and semi-quantification were performed as described by Feng et al. [29]. In order to assess the contribution of specific compounds to the wine aroma, the odor activity value (OAV) was used. We assumed that odorants with higher OAVs > 1 contributed more to the overall aroma.

### 3.7. Sensory Analysis

The tasting sessions took place in a sensory room designed according to the ISO 8589:2010 Standard [73]. The sensory panel comprised 16 expert tasters (9 males and 7 females, 25–50 years old) from the D.O.Ca Rioja. The ISO 11035 method [66] was employed for the selection of descriptors. Before the sensory analysis, the tasters established a shared set of attributes for the visual, olfactory, and gustatory phases. Kt, Mo, Oh, and Py wines were tasted separately for each wine cellar. A structured numerical scale ranging from 0 (absence) to 5 (maximum presence) was employed to evaluate the attributes. 

The geometric mean (GM%) was calculated to classify the descriptors, allowing for the elimination of descriptors with relatively low geometric means. The GM% is calculated as the square root of the product between the relative intensity (I%) and the relative frequency (F%) according to ISO 11035 [66].

Finally, a preference sensory test was carried out. All expert tasters ordered the wines according to their preference for each winery separately, with 1 being the most liked and 4 considered the least preferred. All preference data are expressed as the mean value of the 16 tasters.

### 3.8. Statistical Analyses

The SPSS Version 23.0 statistical package for Windows (IBM Corp., Armonk, NY, USA) was employed to perform the statistical analysis of the data. A multivariate analysis of variance (MANOVA) was conducted on wine samples to determine differences in the analytical parameters among barrel origins.

## 4. Conclusions

Given the extended aging periods typical of high-quality red wines in Spain, it is essential to understand how the use of barrels made of American oak from different geographical areas in the United States influences the chemical and sensory characteristics of the wines over extended periods. Therefore, this work evaluated the impact of *Q. alba* oak from four distinct geographical regions in the United States [Missouri (Mo), Ohio (Oh), Kentucky (Kt), and Pennsylvania (Py)] on the volatile and phenolic composition, as well as the sensory characteristics of Tempranillo wines aged for 12 and 24 months. The influence of the different wood geographical origins on wine was higher on the aromatic composition than on the polyphenolic one. In fact, the results suggested that the specific origin of the oak provided different aromatic profiles depending on the time of aging. For 12 months of aging, Mo and Oh barrels could be recommended to enhance spicy and sweet notes in wines, whereas for 24 months of aging, Kt barrels could be the preferred choice to achieve a complex aromatic profile. Aging in Py barrels produced wines with the lowest content of volatile and phenolic compounds at both aging times, probably due to a higher wood porosity. The barrel and aging time also showed an influence on the phenolic composition of the wines. At 12 months of aging, the wines aged in Kt and Mo barrels exhibited the highest content of ellagitannins and anthocyanins, respectively. However, for a prolonged aging period of 24 months, Oh barrels allowed for higher concentrations of phenolic compounds. In the sensory analysis, Kt wines were preferred throughout the aging process. Kt and Mo wines achieved the highest scores for the olfactory phase at 12 months, with Kt wines maintaining it after 24 months. In the gustatory phase, Kt wines were perceived to have better balance at both aging times.

These results are particularly useful for winemakers, as they provide a better knowledge of the contribution of the oak to the wine according to the different areas in which the trees have grown, as well as the aging time, so that they can choose one area or another depending on the wine to be made.

## Figures and Tables

**Table 1:** Common oenological parameters of the Tempranillo wines after 12 and 24 months of aging.

	12 Months of Aging				24 Months of Aging			
	Kt	Mo	Oh	Py	Kt	Mo	Oh	Py
pH	3.63	3.62	3.63	3.63	3.72	3.71	3.73	3.73
TA	4.97	5.10	5.06	4.98	4.76	4.76	4.81	4.73
VA	0.51 b	0.50 b	0.52 b	0.47 a	0.63 b	0.62 ab	0.63 b	0.59 a
Ethanol	14.00 b	13.82 a	13.79 a	13.92 ab	14.12	13.98	13.93	14.11
CI	14.09	14.16	14.23	14.01	12.22 b	12.16 ab	12.65 c	12.01 a
TPI	73.72 a	77.76 b	74.63 a	74.19 a	63.24 b	63.30 b	61.90 a	63.11 b
T	0.72	0.73	0.72	0.71	0.84	0.84	0.83	0.83
420 nm	0.51	0.51	0.51	0.50	0.49 a	0.49 a	0.51 b	0.48 a
520 nm	0.71	0.71	0.72	0.71	0.59 a	0.58 a	0.61 b	0.58 a
620 nm	0.20	0.20	0.20	0.19	0.14	0.14	0.15	0.14

Kt: wines aged in barrels from Kentucky, Mo: wines aged in barrels from Missouri, Oh: wines aged in barrels from Ohio, Py: wines aged in barrels from Pennsylvania. TA: total acidity (g/L of tartaric acid); VA: volatile acidity (g/L of acetic acid); Ethanol: mL of ethanol per 100 mL of wine at 20 °C (% Vol.); CI: color intensity; TPI: total polyphenol index; T: tonality. Mean values of the wines from the 3 wineries. At the same aging time, different letters in the same row indicate significant differences (*p* ≤ 0.05). The 12-month data and 24-month data were not statistically compared. The absence of letters indicates no significant differences (*p* > 0.05).

**Table 2:** Concentrations (mg/L) of phenolic compounds in Tempranillo wines after 12 months of aging.

	Kt	Mo	Oh	Py
*Hydroxybenzoic acids*				
Gallic acid	88.40 b	89.94 b	86.97 b	81.59 a
Syringic acid	4.22 c	3.51 b	2.76 a	3.57 b
Ellagic acid	1.92 ab	1.97 b	2.33 c	1.84 a
Total	94.54 b	95.42 b	92.06 b	87.00 a
*Hydroxycinnamic acids*				
Caftaric acid	33.22 b	33.52 b	34.17 b	31.66 a
Fertaric acid	5.31	5.14	5.08	5.35
Coutaric acid	28.71 b	27.75 ab	28.49 b	26.90 a
Caffeic acid	7.81 bc	7.17 a	8.07 c	7.61 b
Coumaric acid	2.83 ab	2.94 b	2.96 b	2.72 a
Total	77.88 b	76.53 ab	78.78 b	74.25 a
*Flavonols*				
Myricetin-3-gal	1.84 b	2.03 c	1.54 a	1.88 b
Myricetin-3-glc	12.88 b	13.02 b	13.21 b	11.93 a
Quercetin-3-gal	3.52 b	3.22 a	3.64 b	3.32 a
Quercetin-3-glc	3.55	3.54	3.51	3.39
Quercetin-3-glcU	2.68 ab	2.56 a	2.82 c	2.78 bc
Isorhamnetin-3-glc	1.57 ab	1.58 ab	1.62 b	1.53 a
Myricetin	9.54 ab	9.70 b	9.68 b	9.19 a
Quercetin	4.79 ab	4.67 ab	4.86 b	4.61 a
Kaempferol	0.77 c	0.70 a	0.75 bc	0.73 ab
Isorhamnetin	0.36 c	0.35 b	0.38 d	0.33 a
Total	41.52 ab	41.37 ab	42.01 b	39.70 a
*Flavan-3-ol*				
Catechin	29.93	29.29	29.32	28.77
*Stilbenes*				
*trans*-Piceid	1.38	1.40	1.40	1.36
*trans*-Resveratrol	1.05 c	0.98 b	1.10 c	0.91 a
Total	2.43 b	2.39 ab	2.49 b	2.27 a
*Ellagitannins*	10.95 c	10.10 b	10.34 b	9.08 a
*Anthocyanins*				
Delphinidin-3-glc	49.82 bc	51.82 c	48.53 ab	46.49 a
Cyanidin-3-glc	3.38 b	3.82 c	4.16 d	3.05 a
Petunidin-3-glc	42.05 a	44.38 b	42.46 ab	40.95 a
Peonidin-3-glc	9.80 a	10.42 b	10.19 ab	9.92 a
Malvidin-3-glc	130.87 a	137.18 b	130.60 a	126.79 a
Delphinidin-3-acglc	1.64 b	1.73 c	1.84 d	1.56 a
Cyanidin-3-acglc	1.34	1.33	1.29	1.32
Petunidin-3-acglc	1.79 b	1.67 a	2.03 c	1.70 a
Peonidin-3-acglc	0.65 b	0.60 a	0.61 a	0.84 c
Malvidin-3-acglc	7.39 ab	7.52 b	7.07 a	7.22 ab
Delphinidin-3-cmglc	4.30 a	4.61 b	4.37 a	4.17 a
Cyanidin-3-cmglc	0.88 b	0.80 a	0.87 b	0.91 b
Petunidin-3-cmglc	4.22 ab	4.39 b	4.08 a	4.32 b
Peonidin-3-cmglc	1.73 b	2.03 c	1.61 a	1.75 b
Malvidin-3-cmglc	15.38 a	16.41 b	15.43 a	14.80 a
Total	275.25 a	288.70 b	275.16 a	265.79 a

Kt: wines aged in barrels from Kentucky, Mo: wines aged in barrels from Missouri, Oh: wines aged in barrels from Ohio, Py: wines aged in barrels from Pennsylvania. Mean values of the wines from the 3 wineries. Different letters in the same line indicate statistically significant differences (*p* ≤ 0.05). The absence of letters indicates no significant differences (*p* > 0.05). glc: glucoside, acglc: acetyl-glucoside, cmglc: coumaroyl-glucoside, gal: galactoside, glcU: glucuronide.

**Table 3:** Concentrations (mg/L) of phenolic compounds in Tempranillo wines after 24 months of aging.

	Kt	Mo	Oh	Py
*Hydroxybenzoic acids*				
Gallic acid	37.06	36.86	36.09	35.88
Syringic acid	3.14	3.12	3.12	3.16
Ellagic acid	4.47 b	4.38 b	3.68 a	4.40 b
Total	44.67	44.36	42.90	43.44
*Hydroxycinnamic acids*				
Caftaric acid	47.13 b	46.64 ab	47.92 b	44.50 a
Fertaric acid	3.51 b	3.51 b	3.80 c	3.27 a
Coutaric acid	16.67	16.88	17.52	16.80
Caffeic acid	3.13 a	3.54 b	3.58 b	3.51 b
Ferulic acid	0.33 ab	0.36 c	0.34 bc	0.32 a
Coumaric acid	9.80 d	8.60 c	6.73 b	5.56 a
Total	80.57 b	79.52 b	79.90 b	73.96 a
*Flavonols*				
Myricetin-3-gal	0.48 b	0.42 a	0.56 c	0.43 a
Myricetin-3-glc	4.23	4.21	4.29	4.02
Quercetin-3-gal	0.73 b	0.73 b	0.64 a	0.72 b
Quercetin-3-glc	0.87 b	0.86 b	0.88 b	0.81 a
Quercetin-3-glcU	13.49 b	13.30 b	13.45 b	12.59 a
Isorhamnetin-3-glc	0.67 b	0.67 b	0.71 c	0.62 a
Myricetin	15.15 a	14.98 a	16.48 b	14.86 a
Quercetin	7.29 b	7.96 c	8.25 c	6.65 a
Kaempferol	0.44 a	0.64 b	0.83 c	0.62 b
Isorhamnetin	0.30 a	0.33 b	0.36 c	0.31 a
Total	43.65 ab	44.11 b	46.44 c	41.62 a
*Flavan-3-ols*				
Catechin	12.12 b	12.33 b	12.12 b	11.30 a
Epicatechin	3.75 b	3.67 b	3.70 b	3.40 a
Total	15.87 b	16.00 b	15.82 b	14.71 a
*Stilbenes*				
*trans*-Piceid	1.55 b	0.81 a	0.78 a	0.80 a
*trans*-Resveratrol	0.20 d	0.19 c	0.18 b	0.15 a
Total	1.75 b	1.00 a	0.96 a	0.94 a
Ellagitannins	11.03 ab	11.39 b	12.32 c	10.40 a
*Anthocyanins*				
Delphinidin-3-glc	6.78 b	7.03 b	7.03 b	5.07 a
Cyanidin-3-glc	0.33 c	0.33 c	0.31 b	0.25 a
Petunidin-3-glc	4.80 b	4.82 b	5.14 c	3.57 a
Peonidin-3-glc	1.22 b	1.19 b	1.19 b	0.89 a
Malvidin-3-glc	20.75 b	20.78 b	21.83 b	15.58 a
Delphinidin-3-acglc	0.38 b	0.39 b	0.45 c	0.28 a
Cyanidin-3-acglc	0.03 b	0.03 c	0.04 d	0.03 a
Petunidin-3-acglc	0.16 b	0.16 bc	0.17 c	0.12 a
Peonidin-3-acglc	0.10 b	0.10 b	0.12 c	0.06 a
Malvidin-3-acglc	1.29 b	1.32 b	1.49 c	0.96 a
Delphinidin-3-cmglc	0.74 b	0.78 b	0.91 c	0.53 a
Cyanidin-3-cmglc	0.15 b	0.15 b	0.15 b	0.10 a
Petunidin-3-cmglc	0.21 b	0.21 b	0.23 c	0.14 a
Peonidin-3-cmglc	0.13 c	0.12 b	0.14 d	0.08 a
Malvidin-3-cmglc	1.04 b	1.03 b	1.13 c	0.73 a
Total	38.11 b	38.46 bc	40.34 c	28.39 a

Kt: wines aged in barrels from Kentucky, Mo: wines aged in barrels from Missouri, Oh: wines aged in barrels from Ohio, Py: wines aged in barrels from Pennsylvania. Mean values of the wines from the 3 wineries. Different letters in the same line indicate statistically significant differences (*p* ≤ 0.05). The absence of letters indicates no significant differences (*p* > 0.05). glc: glucoside, acglc: acetyl-glucoside, cmglc: coumaroyl-glucoside, gal: galactoside, glcU: glucuronide.

**Table 4:** Concentration media (μg 4-nonanol/L) of volatile compounds in Tempranillo wines after 12 months of aging.

Compounds	Kt	Mo	Oh	Py
Furanic compounds	1416.97 c	1164.89 b	1186.99 b	811.51 a
Furfural	892.75 c	738.11 b	751.96 b	496.27 a
5-methylfurfural	401.98 c	327.51 b	325.81 b	211.15 a
Furfuryl alcohol	122.24 b	99.28 a	109.22 a	104.09 a
β-methyl-γ-octalactones	838.93 a	1124.06 b	1158.12 b	776.13 a
trans-β-methyl-γ-octalactone	80.21 a	80.40 a	81.77 a	94.45 b
cis-β-methyl-γ-octalactone	758.71 b	1043.67 c	1076.34 c	681.69 a
Volatile phenols	369.63 a	419.36 b	450.49 b	423.16 b
Eugenol	146.68 c	138.53 c	111.60 b	90.98 a
Guaiacol	40.09 a	39.58 a	63.47 b	45.79 a
4-ethylguaiacol	35.35 b	23.65 a	42.31 c	54.26 d
4-ethylphenol	147.51 a	217.61 b	233.11 b	232.13 b
Phenolic aldehydes	251.32	249.93	254.37	281.27
Vanillin	251.32	249.93	254.37	281.27

Kt: wines aged in barrels from Kentucky, Mo: wines aged in barrels from Missouri, Oh: wines aged in barrels from Ohio, Py: wines aged in barrels from Pennsylvania. Mean values of the wines from the 3 wineries. Different letters in the same line indicate statistically significant differences (*p* ≤ 0.05). The absence of letters indicates no significant differences (*p* > 0.05).

**Table 5:** Odor activity values (OAV) of Tempranillo wines after 12 months of aging.

Compound	Odor Threshold (μg/L)	Descriptor	Ref.	12 Months			
				Kt	Mo	Oh	Py
Furanic Compounds							
Furfural	14,100	Burned almonds, incense	[36]	0.06 c	0.05 b	0.05 b	0.04 a
5-methylfurfural	20,000	Bitter almond, spice	[37]	0.02 c	0.02 b	0.02 b	0.01 a
Furfuryl alcohol	15,000	Hay	[38]	0.01 b	0.01 a	0.01 a	0.01 a
β-methyl-γ-octalactones							
cis-β-methyl-γ-octalactone	46	Woody, coconut, vanilla	[39]	16.49 b	22.69 c	23.40 c	14.82 a
trans-β-methyl-γ-octalactone	370	Woody, coconut, vanilla	[40]	0.22 a	0.22 a	0.22 a	0.26 b
Volatile phenols							
Eugenol	6	Clove, honey, spicy	[36]	24.45 c	23.09 c	18.60 b	15.16 a
Guaiacol	9.5	Smoke, toasted, spicy	[36]	4.22 a	4.17 a	6.68 b	4.82 a
4-Ethylguaiacol	140	Toasted, smoky, spicy	[41]	0.25 b	0.17 a	0.30 c	0.39 d
4-Ethylphenol	620	Leather, animal	[41]	0.24 a	0.35 b	0.38 b	0.37 b
Phenolic aldehydes							
Vanillin	200	Vanilla	[42]	1.26	1.25	1.27	1.41
Total OAV of wood-related aromas				47.21 b	52.00 c	50.93 c	37.28 a

Kt: wines aged in barrels from Kentucky, Mo: wines aged in barrels from Missouri, Oh: wines aged in barrels from Ohio, Py: wines aged in barrels from Pennsylvania. Mean values of the wines from the 3 wineries. Different letters in the same line indicate statistically significant differences (*p* ≤ 0.05). The absence of letters indicates no significant differences (*p* > 0.05).

**Table 6:** Concentration media (μg 4-nonanol/L) of volatile compounds in Tempranillo wines after 24 months of aging.

Compounds	Kt	Mo	Oh	Py
Furanic compounds	448.33 c	400.22 b	396.39 b	362.47 a
Furfural	132.24 b	134.24 b	107.79 a	96.99 a
5-methylfurfural	56.40 b	89.78 c	39.04 a	31.66 a
Furfuryl alcohol	259.26 c	176.20 a	249.55 c	233.82 b
β-methyl-γ-octalactones	1128.14 b	966.54 a	1083.36 b	904.78 a
trans-β-methyl-γ-octalactone	113.33 c	93.05 b	125.39 d	83.28 a
cis-β-methyl-γ-octalactone	1014.81 b	873.49 a	957.98 b	821.51 a
Volatile phenols	712.40 b	373.63 a	855.99 c	884.59 c
Eugenol	202.79 c	157.78 b	137.10 a	134.69 a
Guaiacol	29.77	35.25	34.26	34.60
4-ethylguaiacol	127.90 b	46.63 a	129.64 b	124.67 b
4-ethylphenol	351.94 b	133.98 a	554.99 c	590.63 d
Phenolic aldehydes	280.63 a	310.34 b	323.73 b	307.39 ab
Vanillin	280.63 a	310.34 b	323.73 b	307.39 ab

Kt: wines aged in barrels from Kentucky, Mo: wines aged in barrels from Missouri, Oh: wines aged in barrels from Ohio, Py: wines aged in barrels from Pennsylvania. Mean values of the wines from the 3 wineries. Different letters in the same line indicate statistically significant differences (*p* ≤ 0.05). The absence of letters indicates no significant differences (*p* > 0.05).

**Table 7:** Odor activity values (OAV) of Tempranillo wines after 24 months of aging.

Compound	Odor Threshold (μg/L)	Descriptor	Ref.	Kt	Mo	Oh	Py
Furanic compounds							
Furfural	14,100	Burned almonds, incense	[36]	0.01 b	0.01 b	0.01 a	0.01 a
5-methylfurfural	20,000	Bitter almond, spice	[37]	0.00 b	0.00 c	0.00 a	0.00 a
Furfuryl alcohol	15,000	Hay	[38]	0.02 c	0.01 a	0.02 c	0.02 b
β-methyl-γ-octalactones							
cis-β-methyl-γ-octalactone	46	Woody, coconut, vanilla	[39]	22.06 b	18.99 a	20.83 b	17.86 a
trans- β-methyl-γ-octalactone	370	Woody, coconut, vanilla	[40]	0.31 c	0.25 b	0.34 d	0.23 a
Volatile phenols							
Eugenol	6	Clove, honey, spicy	[36]	33.80 c	26.30 b	22.85 a	22.45 a
Guaiacol	9.5	Smoke, toasted, spicy	[36]	3.13	3.71	3.61	3.64
4-Ethylguaiacol	140	Toasted, smoky, spicy	[41]	0.91 b	0.33 a	0.93 b	0.89 b
4-Ethylphenol	620	Leather, animal	[41]	0.57 b	0.22 a	0.89 c	0.95 d
Phenolic aldehydes							
Vanillin	200	Vanilla	[42]	1.40 a	1.55 b	1.62 b	1.54 ab
Total OAV of wood-related aromas				62.21 c	51.37 b	51.08 b	47.58 a

Kt: wines aged in barrels from Kentucky, Mo: wines aged in barrels from Missouri, Oh: wines aged in barrels from Ohio, Py: wines aged in barrels from Pennsylvania. Mean values of the wines from the 3 wineries. Different letters in the same line indicate statistically significant differences (*p* ≤ 0.05). The absence of letters indicates no significant differences (*p* > 0.05).

## Data Availability

Data are contained within the article.

## References

[1] Jordão A.M., Cosme F. (2022). The Application of Wood Species in Enology: Chemical Wood Composition and Effect on Wine Quality. Appl. Sci..

[2] Michel J., Jourdes M., Silva M.A., Giordanengo T., Mourey N., Teissedre P.L. (2011). Impact of concentration of ellagitannins in oak wood on their levels and organoleptic influence in red wine. J. Agric. Food Chem..

[3] Zhang B., Cai J., Duan C.Q., Reeves M.J., He F. (2015). A review of polyphenolics in oak woods. Int. J. Mol. Sci..

[4] Zhang X.K., He F., Zhang B., Reeves M.J., Liu Y., Zhao X., Duan C.Q. (2018). The effect of prefermentative addition of gallic acid and ellagic acid on the red wine color, copigmentation and phenolic profiles during wine aging. Food Res. Int..

[5] Carpena M., Pereira A.G., Prieto M.A., Simal-Gandara J. (2020). Wine aging technology: Fundamental role of wood barrels. Foods.

[6] Ribéreau-Gayon P., Glories Y., Maujean A., Dubourdieu D., Ribéreau-Gayon P., Glories Y., Maujean A., Dubourdieu D. (2006). Aging Red Wines in Vat and Barrel: Phenomena Occurring during Aging. Handbook of Enology.

[7] Sanz M., De Simón B.F., Cadahía E., Esteruelas E., Muñoz Á.M., Hernández M.T., Estrella I. (2012). Polyphenolic profile as a useful tool to identify the wood used in wine aging. Anal. Chim. Acta.

[8] Laqui-Estaña J., López-Solís R., Peña-Neira Á., Medel-Marabolí M., Obreque-Slier E. (2019). Wines in contact with oak wood: The impact of the variety (Carménère and Cabernet Sauvignon), format (barrels, chips and staves), and aging time on the phenolic composition. J. Sci. Food Agric..

[9] De Rosso M., Cancian D., Panighel A., Dalla Vedova A., Flamini R. (2009). Chemical Compounds Released from Five Different Woods Used to Make Barrels for Aging Wines and Spirits: Volatile Compounds and Polyphenols. Wood Sci. Technol..

[10] De Rosso M., Panighel A., Vedova A.D., Stella L., Flamini R. (2009). Changes in chemical composition of a red wine aged in acacia, cherry, chestnut, mulberry, and oak wood barrels. J. Agric. Food Chem..

[11] Tavares M., Jordão A.M., Ricardo-Da-Silva J.M. (2017). Impact of cherry, acacia and oak chips on red wine phenolic parameters and sensory profile. Oeno One.

[12] Rubio-Bretón P., Garde-Cerdán T., Martínez J. (2018). Use of Oak Fragments During the Aging of Red Wines. Effect on the Phenolic, Aromatic, and Sensory Composition of Wines as a Function of the Contact Time with the Wood. Beverages.

[13] Martínez-Gil A.M., del Alamo-Sanza M., Gutiérrez-Gamboa G., Moreno-Simunovic Y., Nevares I. (2018). Volatile composition and sensory characteristics of Carménère wines macerating with Colombian (*Quercus humboldtii*) oak chips compared to wines macerated with American (*Q. alba*) and European (*Q. petraea*) oak chips. Food Chem..

[14] Gallego L., Del Alamo M., Nevares I., Fernández J.A., De Simón B.F., Cadahía E. (2012). Phenolic Compounds and Sensorial Characterization of Wines Aged with Alternative to Barrel Products Made of Spanish Oak Wood (*Quercus pyrenaica* Willd.). Food Sci. Technol. Int..

[15] González-Centeno M.R., Teissedre P.L., Chira K. (2021). Impact of oak wood ageing modalities on the (non)-volatile composition and sensory attributes of red wines. Oeno One.

[16] Fernández De Simón B., Cadahía E., Sanz M., Poveda P., Perez-Magariño S., Ortega-Heras M., González-Huerta C. (2008). Volatile compounds and sensorial characterization of wines from four spanish denominations of origin, aged in Spanish Rebollo (*Quercus pyrenaica* Willd.) oak wood barrels. J. Agric. Food Chem..

[17] Gombau J., Cabanillas P., Mena A., Pérez-Navarro J., Ramos J., Torner A., Fort F., Gómez-Alonso S., García-Romero E., Canals J.M. (2022). Comparative study of volatile substances and ellagitannins released into wine by *Quercus pyrenaica*, *Quercus petraea* and *Quercus alba* barrels. Oeno One.

[18] Navarro M., Kontoudakis N., Gómez-Alonso S., García-Romero E., Canals J.M., Hermosín-Gutíerrez I., Zamora F. (2016). Influence of the botanical origin and toasting level on the ellagitannin content of wines aged in new and used oak barrels. Food Res. Int..

[19] Martínez-Gil A.M., del Alamo-Sanza M., Nevares I., Sánchez-Gómez R., Gallego L. (2020). Effect of size, seasoning and toasting level of *Quercus pyrenaica* Willd. wood on wine phenolic composition during maturation process with micro-oxygenation. Food Res. Int..

[20] Martínez J., Cadahía E., Fernández de Simón B., Ojeda S., Rubio P. (2008). Effect of the Seasoning Method on the Chemical Composition of Oak Heartwood to Cooperage. J. Agric. Food Chem..

[21] Michel J., Jourdes M., Le Floch A., Giordanengo T., Mourey N., Teissedre P.L. (2013). Influence of wood barrels classified by NIRS on the ellagitannin content/composition and on the organoleptic properties of wine. J. Agric. Food Chem..

[22] Jourdes M., Michel J., Saucier C., Quideau S., Teissedre P.-L. (2011). Identification, amounts, and kinetics of extraction of C-glucosidic ellagitannins during wine aging in oak barrels or in stainless steel tanks with oak chips. Anal. Bioanal. Chem..

[23] Fernández de Simón B., Cadahía E., Jalocha J. (2003). Volatile compounds in a Spanish red wine aged in barrels made of Spanish, French, and American oak wood. J. Agric. Food Chem..

[24] Fernández de Simón B., Hernández T., Cadahía E., Dueñas M., Estrella I. (2003). Phenolic compounds in a Spanish red wine aged in barrels made of Spanish, French and American oak wood. Eur. Food Res. Technol..

[25] Garde-Cerdán T., Goñi D.T., Azpilicueta C.A. (2004). Accumulation of volatile compounds during ageing of two red wines with different composition. J. Food Eng..

[26] Guld Z.S., Rácz A., Tima H., Kállay M., Nyitrainé Sárdy D. (2019). Effects of aging in oak barrels on the trans-resveratrol and anthocyanin concentration of red wines from Hungary. Acta Aliment..

[27] Castro-Vázquez L., Alañón M.E., Calvo E., Cejudo M.J., Díaz-Maroto M.C., Pérez-Coello M.S. (2011). Volatile compounds as markers of ageing in Tempranillo red wines from La Mancha D.O. stored in oak wood barrels. J. Chromatogr. A.

[28] Martínez J. (2004). Incidencia del Origen de la Madera de Roble en la Calidad de los Vinos de Tempranillo de la D.O.Ca. Rioja Durante la Crianza en Barrica. Ph.D. Thesis.

[29] Feng Z., Martínez-Lapuente L., Ayestarán B., Guadalupe Z. (2023). Volatile and sensory characterization of Tempranillo wines aged in *Quercus alba* oak barrels of different geographical origins in USA. LWT-Food Sci. Technol..

[30] Feng Z., Martínez-Lapuente L., Palacios A., Ayestarán B., Guadalupe Z. (2024). Influence of *Quercus alba* oak geographical origin on the colour characteristics and phenolic composition of Tempranillo wines. Eur. Food Res. Technol..

[31] García S.O. (2012). Nuevos Orígenes de la Madera de Roble Para la Crianza de Vinos Tintos de la D.O.Ca Rioja. Ph.D. Thesis.

[32] Mislata A.M., Puxeu M., Nart E., de Lamo S., Ferrer-Gallego R. (2021). Preliminary study of the effect of cation-exchange resin treatment on the aging of Tempranillo red wines. LWT-Food Sci. Technol..

[33] Cadahía E., Fernández de Simón B., Sanz M., Poveda P., Colio J. (2009). Chemical and Chromatic Characteristics of Tempranillo, Cabernet Sauvignon and Merlot Wines from DO Navarra Aged in Spanish and French Oak Barrels. Food Chem..

[34] Kyraleou M., Kallithraka S., Chira K., Tzanakouli E., Ligas I., Kotseridis Y. (2015). Differentiation of wines treated with wood chips based on their phenolic content, volatile composition, and sensory parameters. J. Food Sci..

[35] Li S.Y., Duan C.Q. (2019). Astringency, Bitterness and Color Changes in Dry Red Wines before and during Oak Barrel Aging: An Updated Phenolic Perspective Review. Crit. Rev. Food Sci. Nutr..

[36] Ferreira V., López R., Cacho J.F. (2000). Quantitative determination of the odorants of young red wines from different grape varieties. J. Sci. Food Agric..

[37] Etiévant P.X., Maarse H. (1991). Wine. Volatile Compounds in Foods and Beverages.

[38] Cutzach I., Chatonnet P., Henry R., Pons M., Dubourdieu D. (1998). Study in aroma of sweet natural non-muscat wines 2nd part: Quantitative analysis of volatil compounds taking part in aroma of sweet natural wines during ageing. J. Int. Sci. Vigne Vin..

[39] Wilkinson K.L., Elsey G.M., Prager R.H., Tanaka T., Sefton M.A. (2004). Precursors to oak lactone. Part 2: Synthesis, separation, and cleavage of several β-D-glucopyranosides of 3-methyl-4-hydroxyoctanoic acid. Tetrahedron.

[40] Brown R.C., Sefton M.A., Taylor D.K., Elsey G.M. (2006). An odour detection threshold determination of all four possible stereoisomers of oak lactone in a white and a red wine. Aust. J. Grape Wine Res..

[41] Chatonnet P., Dubourdie D., Boidron J.N., Pons M. (1992). The origin of ethylphenols in wines. J. Sci. Food Agric..

[42] Guth H. (1997). Quantitation and sensory studies of character impact odorants of different white wine varieties. J. Agric. Food Chem..

[43] Chira K., Teissedre P.L. (2013). Extraction of oak volatiles and ellagitannin compounds and sensory profile of wine aged with French winewoods subjected to different toasting methods: Behaviour during storage. Food Chem..

[44] Lu H., Cheng B., Lan Y., Duan C., He F. (2024). Modifications in aroma characteristics of ‘Merlot’ dry red wines aged in American, French, and Slovakian oak barrels with different toasting degrees. Food Sci. Hum. Wellness.

[45] Quijada-Morín N., Dangles O., Rivas-Gonzalo J.C., Escribano-Bailón M.T. (2010). Physico-chemical and chromatic characterization of malvidin 3-glucoside-vinylcatechol and malvidin 3-glucoside-vinylguaiacol wine pigments. J. Agric. Food Chem..

[46] Waterhouse A.L., Towey J.P. (1994). Oak Lactone Isomer Ratio Distinguishes between Wine Fermented in American and French Oak Barrels. J. Agric. Food Chem..

[47] Atanasova V., Fulcrand H., Cheynier V., Moutounet M. (2002). Effect of oxygenation on polyphenol changes occurring in the course of wine-making. Anal. Chim. Acta.

[48] Es-Safi N.E., Cheynier V., Moutounet M. (2002). Role of aldehydic derivatives in the condensation of phenolic compounds with emphasis on the sensorial properties of fruit-derived foods. J. Agric. Food Chem..

[49] Fernandes A., Oliveira J., Teixeira N., Mateus N., de Freitas V. (2017). A review of the current knowledge of red wine colour. Oeno One.

[50] Alañón M.E., Marchante L., Alarcón M., Díaz-Maroto I.J., Pérez-Coello S., Díaz-Maroto M.C. (2018). Fingerprints of acacia aging treatments by barrels or chips based on volatile profile, sensorial properties, and multivariate analysis. J. Sci. Food Agric..

[51] Del Alamo Sanza M., Nevares Domínguez I., Cárcel Cárcel L.M., Navas Gracia L. (2004). Analysis for Low Molecular Weight Phenolic Compounds in a Red Wine Aged in Oak Chips. Anal. Chim. Acta.

[52] Cadahía E., Muñoz L., De Simón B.F., García-Vallejo M.C. (2001). Changes in Low Molecular Weight Phenolic Compounds in Spanish, French, and American Oak Woods During Natural Seasoning and Toasting. J. Agric. Food Chem..

[53] Ginjom I., D’Arcy B., Caffin N., Gidley M. (2011). Phenolic Compound Profiles in Selected Queensland Red Wines at All Stages of the Wine-Making Process. Food Chem..

[54] Matějíček D., Mikeš O., Klejdus B., Štěrbová D., Kubáň V. (2005). Changes in Contents of Phenolic Compounds during Maturing of Barrique Red Wines. Food Chem..

[55] Sanz M., Fernández de Simón B., Esteruelas E., Muñoz Á.M., Cadahía E., Hernández M.T., Estrella I., Martinez J. (2012). Polyphenols in Red Wine Aged in Acacia (*Robinia pseudoacacia*) and Oak (*Quercus petraea*) Wood Barrels. Anal. Chim. Acta.

[56] Chinnici F., Natali N., Bellachioma A., Versari A., Riponi C. (2015). Changes in Phenolic Composition of Red Wines Aged in Cherry Wood. LWT-Food Sci. Technol..

[57] Martínez-Gil A., Del Alamo-Sanza M., Nevares I. (2022). Evolution of Red Wine in Oak Barrels with Different Oxygen Transmission Rates. Phenolic Compounds and Colour. LWT-Food Sci. Technol..

[58] Zhang Q.A., Wang T.T. (2017). Effect of Ultrasound Irradiation on the Evolution of Color Properties and Major Phenolic Compounds in Wine during Storage. Food Chem..

[59] Gutiérrez I.H., Lorenzo E.S.P., Espinosa A.V. (2005). Phenolic Composition and Magnitude of Copigmentation in Young and Shortly Aged Red Wines Made from the Cultivars, Cabernet Sauvignon, Cencibel, and Syrah. Food Chem..

[60] Barrera-García V.D., Gougeon R.D., Di Majo D., De Aguirre C., Voilley A., Chassagne D. (2007). Different Sorption Behaviors for Wine Polyphenols in Contact with Oak Wood. J. Agric. Food Chem..

[61] Espitia-López J., Escalona-Buendía H.B., Luna H., Verde-Calvo J.R. (2015). Multivariate Study of the Evolution of Phenolic Composition and Sensory Profile on Mouth of Mexican Red Merlot Wine Aged in Barrels vs Wood Chips. CyTA-J. Food.

[62] Chira K., Teissedre P.L. (2015). Chemical and Sensory Evaluation of Wine Matured in Oak Barrel: Effect of Oak Species Involved and Toasting Process. Eur. Food Res. Technol..

[63] Jordão A.M., Ricardo-Da-Silva J.M., Laureano O. (2007). Ellagitannins from Portuguese Oak Wood (*Quercus pyrenaica* Willd.) Used in Cooperage: Influence of Geographical Origin Coarseness of the Grain and Toasting Level. Holzforschung.

[64] Chinnici F., Natali N., Sonni F., Bellachioma A., Riponi C. (2011). Comparative Changes in Color Features and Pigment Composition of Red Wines Aged in Oak and Cherry Wood Casks. J. Agric. Food Chem..

[65] Gambuti A., Capuano R., Lisanti M.T., Strollo D., Moio L. (2010). Effect of Aging in New Oak, One-Year-Used Oak, Chestnut Barrels and Bottle on Color, Phenolics and Gustative Profile of Three Monovarietal Red Wines. Eur. Food Res. Technol..

[66] (1994). Sensory Analysis. Identification and Selection of Descriptors for Establishing a Sensory Profile.

[67] OIV (2003). Compendium of Internationals Methods of Wine and Must Analysis.

[68] Ribéreau-Gayon P., Glories Y., Maujean A., Dubourdieu D. (2000). The chemistry of wines, stabilization and treatments. Handbook of Enology.

[69] Gómez-Alonso S., García-Romero E., Hermosín-Gutiérrez I. (2007). HPLC analysis of diverse grape and wine phenolics using direct injection and multidetection by DAD and fluorescence. J. Food Compos. Anal..

[70] Peng S., Scalbert A., Monties B. (1991). Insoluble ellagitannins in Castanea sativa and *Quercus petraea* woods. Phytochemistry.

[71] Oliveira J.M., Faria M., Sá F., Barros F., Araújo I.M. (2006). C6-alcohols as varietal markers for assessment of wine origin. Anal. Chim. Acta.

[72] Coelho E., Lemos M., Genisheva Z., Domingues L., Vilanova M., Oliveira J.M., Vilanova M., Oliveira J.M. (2020). Validation of a LLME/GC-MS methodology for quantification of volatile compounds in fermented beverages. Molecules.

[73] (2010). Sensory Analysis—General Guidance for the Design of Test Rooms.

